# Evaluation of anticancer potential of Thai medicinal herb extracts against cholangiocarcinoma cell lines

**DOI:** 10.1371/journal.pone.0216721

**Published:** 2019-05-23

**Authors:** Bundit Promraksa, Jutarop Phetcharaburanin, Nisana Namwat, Anchalee Techasen, Patcharee Boonsiri, Watcharin Loilome

**Affiliations:** 1 Department of Biochemistry, Faculty of Medicine, Khon Kaen University, Khon Kaen, Thailand; 2 Cholangiocarcinoma Research Institute, Faculty of Medicine, Khon Kaen University, Khon Kaen, Thailand; 3 Center of Excellence for Innovation in Chemistry, Mahidol University, Bangkok, Thailand; 4 Faculty of Associated Medical Science, Khon Kaen University, Khon Kaen, Thailand; Duke University School of Medicine, UNITED STATES

## Abstract

Although cholangiocarcinoma (CCA) has a low incidence globally, this is extremely high in Northeast Thailand. The lack of both early detection measures and effective therapeutic drugs is the major problem for the poor prognosis of CCA patients. Based on regional knowledge, it would be advantageous to search for effective natural phyto-products for the treatment of CCA. *Cardiospermum halicacabum* L., *Gomphrena celosioides* Mart. and *Scoparia dulcis* L., very well-known medicinal herbs in Asian countries, were selected for the investigation of inhibitory effects on CCA cells. Of the three different ethanolic extracts, *S*. *dulcis* L extract showed most inhibitory effects on cell growth of CCA cell lines KKU-100 and KKU-213, at percentages of 56.06 and 74.76, respectively, compared to the untreated group after treatment with 250 μg/mL of extracts for 72 hrs. At 400 and 500 μg/mL of the extracts, the inhibitory effect of KKU-213 was indicated by a significant increase in the BAX/Bcl-2 ratio and cell membrane permeability. Moreover, metabolic profiling-based screening employed in the current study revealed a significant positive association between the lignin compound and a decrease in CCA cell viability. Our study suggests, for the first time, that ESD has the ability to inhibit CCA cell growth through the induction of apoptosis.

## Introduction

Cholangiocarcinoma (CCA) is a cancer originating in hepatic biliary epithelial cells. Although this type of cancer has a low incidence in most countries worldwide, it has a very high incidence in the Mekong Region of Southeast Asia, especially in northeast Thailand where it is 87.7 per 100,000 for males and 36.3 for females [1]. Here, host-parasite defense mechanisms contribute to cholangiocarcinogenesis [2]. Free radicals, including reactive oxygen species (ROS) and reactive nitrogen species (RNS), arise from chronic *Opisthorchis viverrini* infection. These can cause severe damage to proteins, nucleic acids and lipids and can potentially lead to CCA initiation and progression [3–5]. As CCA is usually diagnosed at an advanced stage the prognosis is poor and treatment largely ineffective. The lack of early detection and effective therapeutic treatment for this cancer are major reasons for its poor prognosis and control [6]. Treatments of CCA include surgery, radiation- and/or chemotherapy. Many anticancer drugs, such as 5-fluorouracil and gemcitabine, have been frequently used for the treatment of CCA patients, however, there is a low response rate, short median survival time and toxicity, along with a decrease in blood cell production and electrolyte imbalance [7]. At present, several mechanisms for anticancer drug resistance have been proposed, including changes in cellular uptake and efflux of the drug [8, 9]. Therefore, the search for effective therapeutic agents for CCA is necessary.

**Fig 1:**
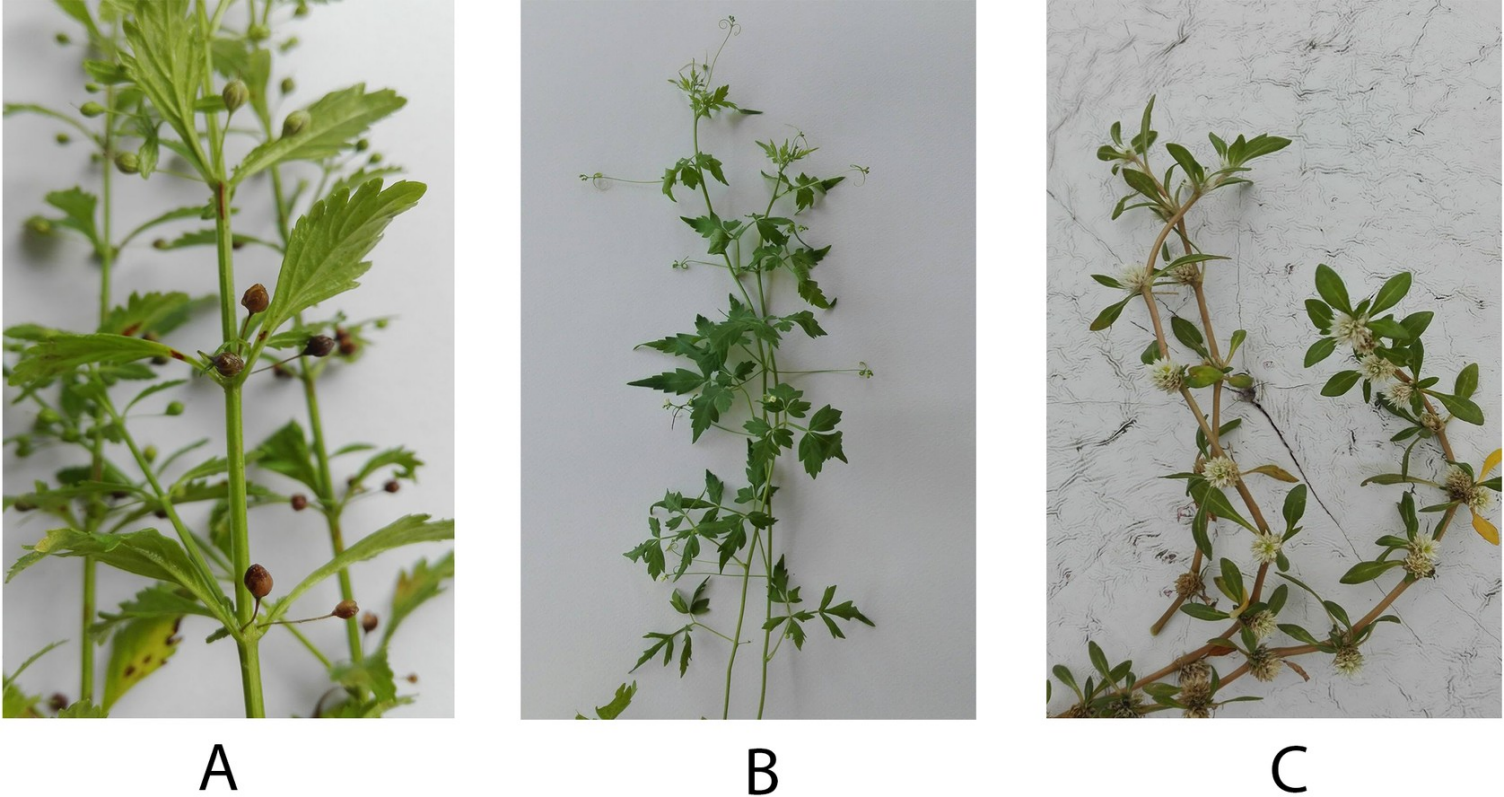
Selected Thai herbs used in the current study. *(A) S*. *dulcis*; (B) *C*. *halicacabum*; (C) *G*. *celosioides*.

The daily diet of a large quantity of vegetables, fruits, and other plant products can reduce the risks for aging-related diseases such as cancer and hypertension, and stabilize blood sugar levels [10]. Because medicinal plants contain non-nutritive metabolites or phytochemicals, these plants are, therefore, becoming an abundant source of such agents for cancer prevention and treatment. Most plant phenolics have antioxidant properties, but they also play a role in cytotoxicity. Many studies suggest that high phenolic content is correlated with high antioxidant activity, and has a cytotoxicity on cancer cells greater than that of low concentrations of phenolics [11]. The use of effective doses of plant phenolics allows the integrative practitioner to increase tumor response to therapy [12]. The local Thai medicinal plants *Cardiospermum halicacabum* L. (balloon vine), *Gomphrena celosioides* Mart. (Soft khaki weed) and *Scoparia dulcis* L. (Sweet Broomwort) are tested for their efficacy in this study (Fig 1). The Ayurvedic use of *C*. *halicacabum* for the treatment of rheumatoid arthritis has been previously reported [13]. *G*. *celosioides* has diuretic potency in addition to relieving liver disease [14, 15], and *S*. *dulcis* can be used as an anti-diabetes and anti-inflammatory agent [16]. Several studies have reported the bioactive compounds of *S*. *dulcis* using different bioassays. A 70% ethanol extract of *S*. *dulcis* contains polyoxygenated flavone as a major compound and has a cytotoxic effect on HeLa cells [17]. Scopadulcis acid B, which is a diterpene suppressing the effects of tumor promoter 12-O-tetradecanoylphorbol-13-acetate (TPA), is considered as the activator for the signal transduction enzyme protein kinase C (PKC), leading to the inhibition of skin tumor formation in mice induced by 7,12-dimethylbenz[a]anthracene [18]. However, the anti-cancer ability of these medicinal plants has been little studied.

Metabolomics provides the entire collection of metabolites in a living system, i.e. the metabolome, which is a complex and multidimensional data set that can be further analyzed using a chemometric approach to discover biological activity-related bioactive compounds [19, 20]. Mass spectrometry (MS) and nuclear magnetic resonance (NMR) spectroscopy are the major analytical platforms in metabolomics research [21]. However, NMR spectroscopy is the more powerful analytical tool for metabolic profiling using two-dimensional techniques for the evaluation of the structures of bioactive compounds derived from plant extracts [22]. Due to the complexity of metabolome data, principal component analysis (PCA) is used to transform the variables of each sample into fewer dimensions [23]. To identify bioactive compounds, orthogonal partial least squares (O-PLS) regression analysis can be employed using continuous predictive information among plant samples to filter out unwanted variables in the data [24, 25]. As a consequence, the metabolic profiling-based screening of medicinal plants reduces the number of steps and bioassays required in the conservative screening method [26]. This leads to an increased interest in the use of metabolomics, together with the chemometric approach, for the identification of bioactive compounds from selected Thai medicinal herbs, especially in anti-CCA activity. Therefore, the aim of this study was to determine whether anti-CCA metabolites were present in ethanolic extracts of *S*. *dulcis*, *C*. *halicacabum* and *G*. *celosioides*.

## Materials and methods

### Plant collection

The selected herbs, *C*. *halicacabum* (ECH), *G*. *celosioides* (EGC) and *S*. *dulcis* (ESD) were harvested from local fields in Phetchabun province, Thailand (16°17'43.5"N 101°05'23.2"E) during January 2016. Taxonomic classification of these plants was conducted by Prof. Dr. Arunrat Chaveerach (Department of Biology, Faculty of Science, Khon Kaen University).

### Chemicals and reagents

For extraction, commercial grade ethanol (S.C. Science Co., Ltd., Thailand) was used. Folin-Ciocalteau, 2,4,6-Tripyridyl-s-triazine (TPTZ), 2,2-Diphenyl-1-picrylhydrazyl (DPPH), Sodium hydroxide (NaOH), (±)-6-Hydroxy-2,5,7,8-tetramethylchromane-2-carboxylic acid (Trolox), gallic acid, Hydrochloric acid (HCl), Sulforhodamine B (SRB) and skim milk were purchased from Sigma-Aldrich (St Louis, MO, USA). Sodium percarbonate (Na_2_CO_4_) was purchased from Carlo Erba (Milan, Italy). Ferric chloride hexahydrate (FeCl_3_·6H_2_O) was purchased from AnalaR (Lutterworth, UK). Polyoxyethylene-20 (Tween 20) was obtained from Bio Basic (Ontario, Canada). Ham's F12 nutrient mixture, bovine serum albumin (BSA), penicillin-streptomycin and trypsin-EDTA were obtained from Life technologies (Grand Island, NY, USA). Enhanced chemiluminescence plus solution (ECL) was provided by GE healthcare (Buckinghamshire, UK). Pierce bicinchoninic acid (BCA) protein assay kit and CM-H_2_DCFDA (General Oxidative Stress Indicator) were provided by Thermo Scientific (Rockford, IL, USA). Potassium dihydrogen phosphate (KH_2_PO_4_) and Deuterium Oxide (D_2_O) were purchased from Merck (Darmstadt, Germany). 3-(Trimethyl-silyl)propionic acid-d4 sodium salt (TSP), used as the internal standard reference for NMR analysis, was obtained from Cambridge Isotope Laboratories (Andover, MA, USA).

The primary antibodies (Ab), including mouse anti-actin Ab, mouse-anti BAX Ab and rabbit-anti Bcl-2 Ab, were purchased from Abcam (Cambridge, UK). The secondary Ab including anti-mouse Ab and anti-rabbit Ab were purchased from GE healthcare (Buckinghamshire, UK) and Sigma (St Louis, MO, USA), respectively.

### Cell lines

The CCA cell lines KKU-100 (JCRB 1568) and KKU-213 (JCRB 1557) were developed by Prof. Banchob Sripa under the Cholangiocarcinoma Research Institute, Khon Kaen University, Thailand. The protocol for the cell development was approved by the Ethics Committee for Human Research, Khon Kaen University *(*#HE571283). The cells were obtained from the Japanese Collection of Research Bioresources (JCRB) Cell Bank, Osaka, Japan and cultured in Ham's F12 nutrient mixture supplemented with 10% fetal bovine serum and 100 IU/mL of penicillin-streptomycin. The cultured cell lines were incubated at 37°C in a humidified incubator with 5% CO_2_ atmosphere.

### Sample preparation and crude extraction

The leaves of herbs were dried in a hot air oven at 50°C. The dried leaves were chopped and mashed into fine powder (100 g). Next, the powder was macerated in one liter of 90% ethanol for five days at room temperature. The liquid extract was evaporated under a vacuum using a rotary evaporator (Buchi, Switzerland). The crude extracts of *C*. *halicacabum* (ECH), *G*. *celosioides* (EGC), *Scoparia dulcis* (ESD) were separately obtained and stored at 4°C for further analyses.

### Determination of total phenolic content

A total of 500 μL of 0.2 N Folin-Ciocalteau reagent was added to 200 μL of the extract. After incubation with light protection for 30 min, 400 μL of 7% Na_2_CO_4_ was added. The absorbance of the mixture was measured at 750 nm using a spectrometer (Genesys 20, Thermo scientific, USA). As compared with the standard compound, TPC was reported as milligrams of gallic acid equivalent per gram dry weight (μg GAE/mg dry wt).

### Antioxidant assessments by ferric reducing antioxidant power (FRAP) assay

This experiment was performed as described in a previous study [27]. FRAP reagent was freshly prepared by mixing 300 mM sodium acetate buffer pH 3.6, 10 mM TPTZ in 40 mM HCl, 20 mM FeCl_3_•6H_2_O at ratio of 10:1:1. Then, 500 μL of FRAP reagent was added to 500 μL of the extract and left at room temperature for 5 min. The absorbance at 593 nm was then recorded. This antioxidant activity was reported as milligrams gallic acid equivalent per gram dry weight (μg GAE/mg dry wt).

### DPPH^•^ radical scavenging activity of selected herb extracts

A total of 1 μg/mL of the extracts and standard Trolox were used in this experiment. Briefly, 80 μL of the extract was added into 120 μL of 200 μM DPPH reagent. After gently shaking for 2 min, the mixture was left for 30 min at room temperature with light protection. After that, the absorbance was measured at 517 nm using a microplate reader (Tecan, Switzerland). The measurement was carried out in triplicate. The calculation was performed as described in a previous report [28].

### Cytotoxicity test

All tested cell lines were seeded into 96-well culture plates at a density of 2×10^3^ cells/well. After culturing for 12 hrs, the media was treated with the extract dissolved in deionized water and further diluted in cultured media. The cells were cultured for a further 48 and 72 hrs. In addition, the plate for no-growth control (day 0) was set and cell viability determined using the SRB assay as previously described [29]. In brief, cultured cells were fixed with 10% trichloroacetic acid for 1 hr. After washing with phosphate buffered saline, fifty microliters of SRB dye was added into each well and incubated for 45 min. Then, 1% acetic acid was used to wash the unbound dye. Plates were dried at 60°C. Two hundred microliters of 10 mM Tris-base pH 10.5 was added into each well to lyse the cells. The absorbance was determined with microplate reader (Tecan, Switzerland). The highest inhibitory effect on CCA among selected herb extracts was selected for further studies.

### Clonogenic assay

The clonogenic assay was performed as described in a previous study [30]. Exponentially growing cells were harvested from a stock culture and seeded at 200 cells/well in six-well plates. After incubation for 12 hrs, the existing media was removed. Cells were treated with 50–250 μg/mL of ESD. The plates were incubated for 48 hrs. After incubation, the existing media was then removed and renewed every 3 days. The colonies were assessed after culturing for 7 days. They were fixed with paraformaldehyde (4.0% v/v) and stained with crystal violet (0.5% w/v). The images of colonies were captured using a camera (taken under uniform conditions). Then, the colony area was determined using imageJ software. The experiment was conducted in duplicate.

### Detection of intracellular reactive oxygen species

Briefly, CCA cells were plated into six-well plates in complete HAM medium for 18 hours. Then, 50–200 μg/mL of crude extract was added into the cell culture for 48 hrs. After that, the cells were washed twice using PBS, then incubated in medium containing 5 μM of CM-H_2_DCFDA. After 20 min, the cells were washed with PBS and the cell pellets collected by trypsinization. Five hundred mL of PI was added before finally examining the intensity of fluorescence by flow cytometry (FACSCanto II, BD Biosciences, UK). The cellular ROS level was determined in duplicate.

### Annexin V/PI staining

Annexin-V and propidium iodide (PI) staining were used to determine apoptotic and necrotic cells. At first, CCA cells (2x10^4^ cell/mL) were plated in the chamber slide (Corning, USA) and incubated for 24 hrs. Then, 100–500 μg/mL of crude extract was added into the cell culture for 48 hrs. To monitor apoptotic and necrotic cells, the media was removed from the cell culture slide. Annexin-V and PI diluted in incubation buffer (each 1:200) were added and incubated for 10–15 min at room temperature. Finally, staining cells were immediately captured using fluorescence microscopy (Zeiss LSM 800, Carl Zeiss, Germany).

### Western blot analysis

CCA cells were treated with various concentrations of the extracts for 48 hrs. After collecting the living cells, these were washed with ice-cold phosphate buffered saline and lysed using RIPA lysis buffer (0.1% sodium dodecyl sulfate (SDS), 0.5% sodium deoxycholate, 50 mM Tris, 1% Tween 20 and protease cocktail inhibitor). The protein concentration was determined using BCA Protein assay. Equal amounts of protein were resolved in 4x SDS buffer and boiled at 95°C. Then, proteins were loaded and separated by 10% (w/v) SDS-polyacrylamide gel electrophoresis before being transferred to a polyvinylidene fluoride membrane (Immobilon, Merck). After blocking the non-specific sites with 5% (w/v) skimmed milk in Tris-buffered saline (TBS), the membrane was probed with primary Ab, mouse anti-actin Ab (1:10,000), mouse-anti-BAX Ab (1:1,000) and rabbit-anti Bcl-2 Ab (1:1,000). Then, the membrane was washed with TBS containing 0.1% Tween 20 (TTBS) 3 times and TBS once before being incubated with secondary Ab conjugated with horseradish peroxidase. Immunodetection was performed using ECL. The apparent density of the bands on membranes was captured using ImageQuantTM Imager (GE Healthcare UK Ltd., UK). The experiments were performed in triplicate.

### ^1^H-NMR based metabolomics analysis of medicinal plants

Twenty mg of plant extract was placed into a test tube. D_2_O (pH 7.0) containing 0.1% (w/w) TSP was added and vortexed for 1 min at room temperature. After 20 min ultrasonication at room temperature, the mixture was passed through a 0.20 μm filter (Corning, USA). The supernatant was transferred into a 5 mm NMR tube. Proton NMR spectra were acquired using a 400 MHz NMR spectrometer (Bruker, USA). Chemical shift referencing, baseline correction and phasing were performed. Next, the NMR spectral data were processed using MestReNova (Mestrelab Research, USA) software to adjust the peak alignment, normalization and scaling. Multivariate statistical analysis was used to identify differences among samples. PCA and O-PLS analyses were performed using SIMCA-P+ version 12 (Umetrics Inc., Sweden). The data were mean-centered and scaled to Pareto. To confirm the assignment of correlated resonances, statistical total correlation spectroscopy (STOCSY) was employed [31]. Moreover, the resonances of interest were searched against online metabolite databases such as biological magnetic resonance data bank (BMRD) and human metabolome database (HMDB) [32]. To further confirm the metabolite assignment, two-dimensional (2-D) NMR experiments, including correlation spectroscopy (COSY) and heteronuclear multiple bond coherence (HMBC), were performed.

### Statistical analysis

The TPC, antioxidant assessments, cytotoxicity test and BAX:Bcl-2 ratio were presented as mean±standard deviation (S.D.) from three independent experiments and analysed using student’s *t* tests in SPSS version 17.0 (SPSS Inc., USA). A p-value <0.05 was considered as statistically significant. Multivariate analysis was performed using MATLAB (Mathworks, USA) and SIMCA-P+ (Umetrics, Sweden) softwares [31].

## Results

### Phenolic content and antioxidant activity assessments

**Fig 2:**
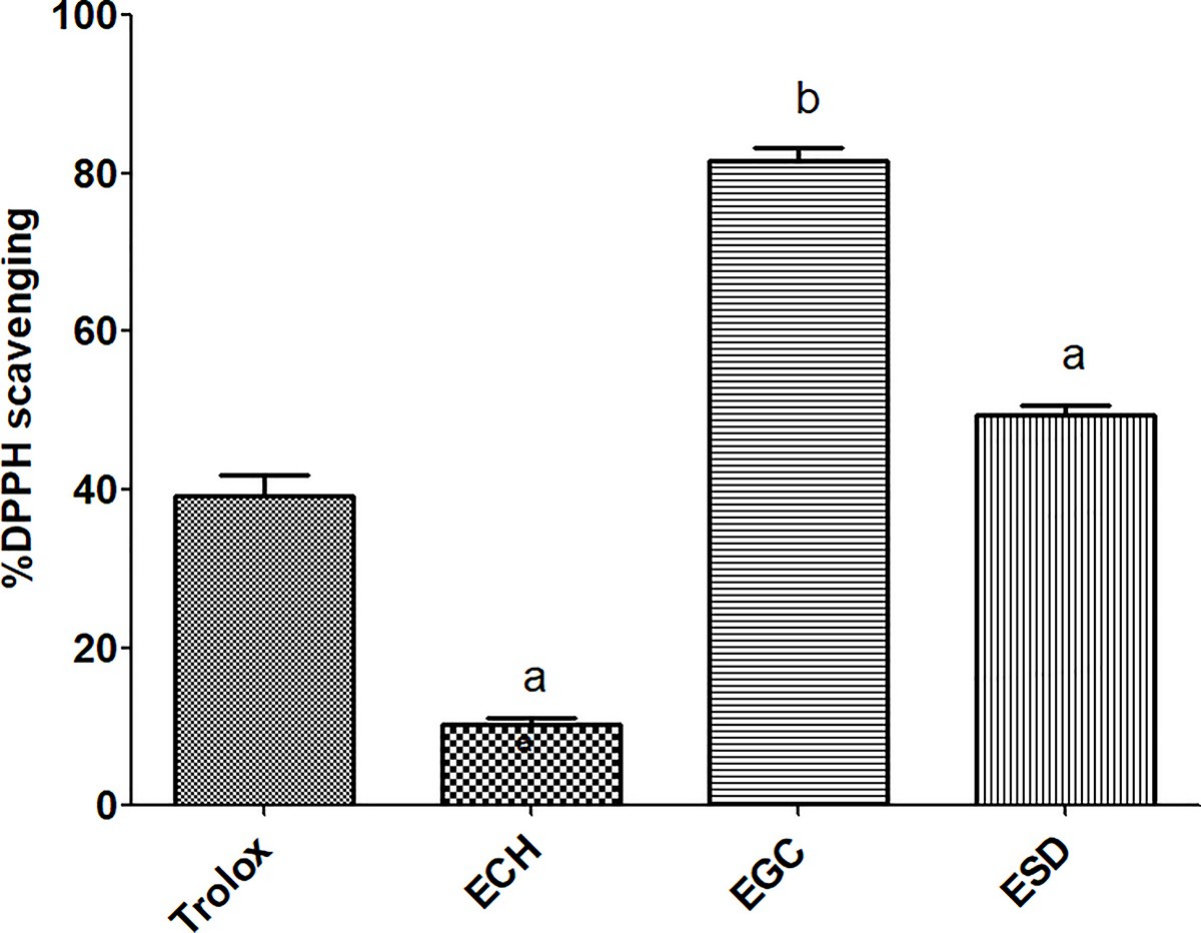
DPPH^•^ radical scavenging activity of selected herb extracts after treatment with 100 μg/mL of each extract and the standard compound (a = p-value <0.05, b = p-value <0.001).

In this study, total phenolic content was measured using the Folin–Ciocalteu method. The linear calibration curve was plotted by the series of gallic acid concentrations against their absorbance. This gave a coefficient of correlation (r) value of >0.95. ESD was shown to contain a slightly higher phenolic content compared with EGC. However, EGC showed the highest antioxidant activity followed by ESD and ECH (Table 1). At a similar concentration, EGC and ESD demonstrated a greater inhibition of the DPPH^**•**^ radical compared with the Trolox standard (Fig 2).

**Table 1:** Total phenolic content and its antioxidant accessed by FRAP method.

Herb extracts	Total phenolic content (μg GAE/mg dry wt)	FRAP assay	
		(μg GAE/mg dry wt)	(μg Trolox/mg dry wt)
ECH	91.13±26.31	9.89±0.06	40.69±0.35
EGC	777.60±23.57	21.59±0.20	109.92±1.15
ESD	784.00±30.53	21.26±0.42	107.97±2.51

### Cytotoxicity test

**Fig 3:**
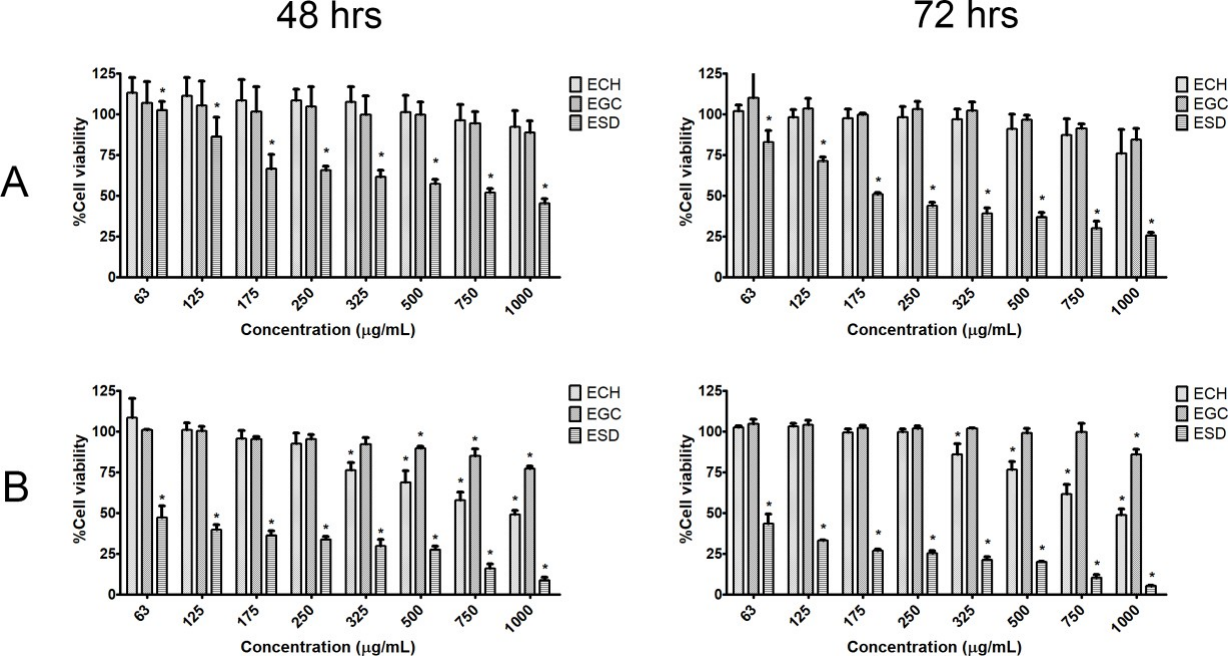
**Percentage of CCA cell viability for (A) KKU-100 and (B) KKU-213 after treatment with each extract for 48 and 72 hrs** (* = statistical significance; p<0.05).

The cell viability of the two CCA cell lines was assessed using SRB assay following 48 and 72 hrs of treatment with plant extracts at 62.5–1,000 μg/mL. The results showed that all CCA cell lines responded to the cytotoxic effects of the plant extract in dose- and time-dependent manners (Fig 3). ESD was shown to have the greatest inhibitory effect on CCA cell growth compared with other plant extracts. After the treatment with 250 μg/mL of extract, only ESD significantly inhibited the growth of KKU-100 and KKU-213 with a percentage of cell viability of 34.45% ± 2.76 and 66.11% ± 1.63%, respectively, after 48 hrs of treatment, and 56.06% ± 2.13 and 74.76% ± 1.64%, respectively, after 72 hrs of treatment. In contrast, ECH and EGC showed only slight inhibitory effects on CCA cell growth even at higher concentrations. Therefore, ESD was selected for further functional analysis. The half maximal inhibitory concentration (IC50) is present in S1 Table.

### Clonogenic assay of ESD

**Fig 4:**
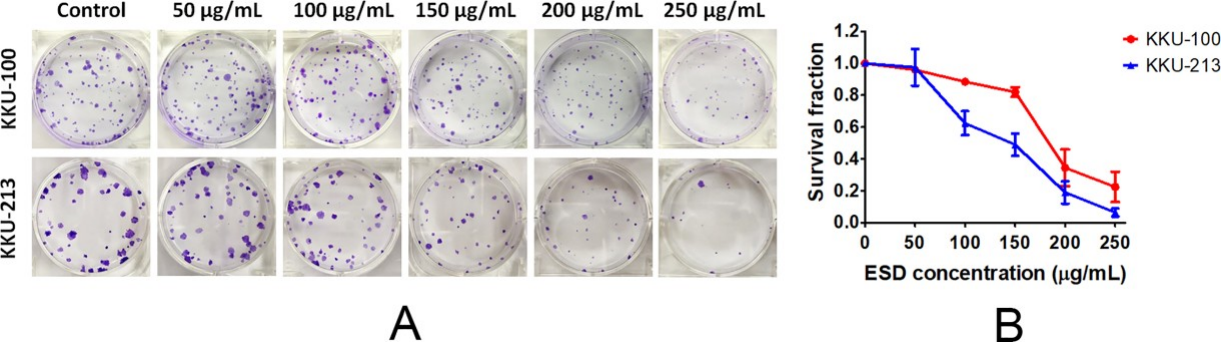
Colony formation assay, the effect of ESD treatment on cancer cells for 48 hrs. Photographs of 6-well plates in a representative experiment are shown in (A). Survival fractions for ESD treatment (B) were normalized by dividing by the survival fractions for untreated cells.

Clonogenic assay is normally used to determine the potential of single cells by colony formation. CCA cell lines were seeded in a culture dish. This method can be employed to observe the drug sensitivity of cancer cells. In Fig 4, the percentage of colony formation declined in ESD in a dose-dependent manner, similarly to the inhibitory effect on CCA cells. The potential inhibition of ESD on KKU-100 cell was less than that on those of KKU-213.

### ESD induced apoptosis effects

**Fig 5:**
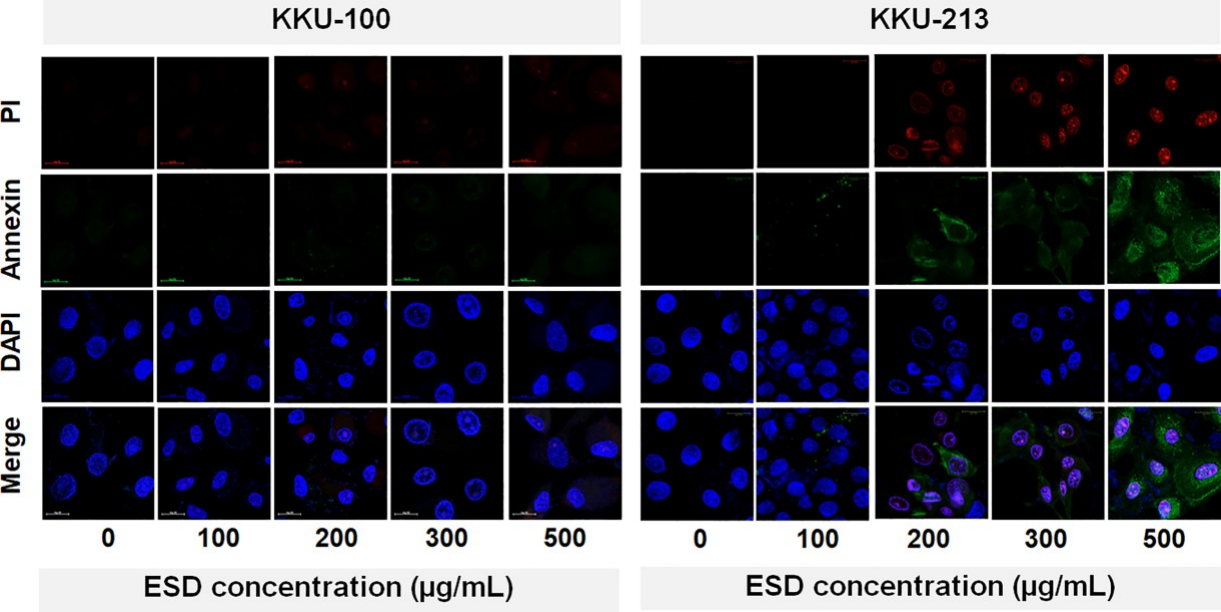
Annexin V/PI staining after ESD treatment for 48 hrs.

ESD induced oxidative stress in CCA cells after treatment for 48 hrs (S1 Fig). The percentage of cellular ROS level in both studied cell lines increased in a dose dependent manner (S2 Fig). Furthermore, the apoptosis induction of the ESD was determined using cell staining with Annexin V and PI. Early apoptotic cells showed the loss of plasma membrane asymmetry which was detected using Annexin V binding with phosphatidylserine. Then, PI binds with DNA in late apoptosis. The apoptotic cells were detected using fluorescence microscopy. ESD induced KKU-213 cell apoptosis, however, apoptotic cells were not observed on KKU-100 (Fig 5).

**Fig 6:**
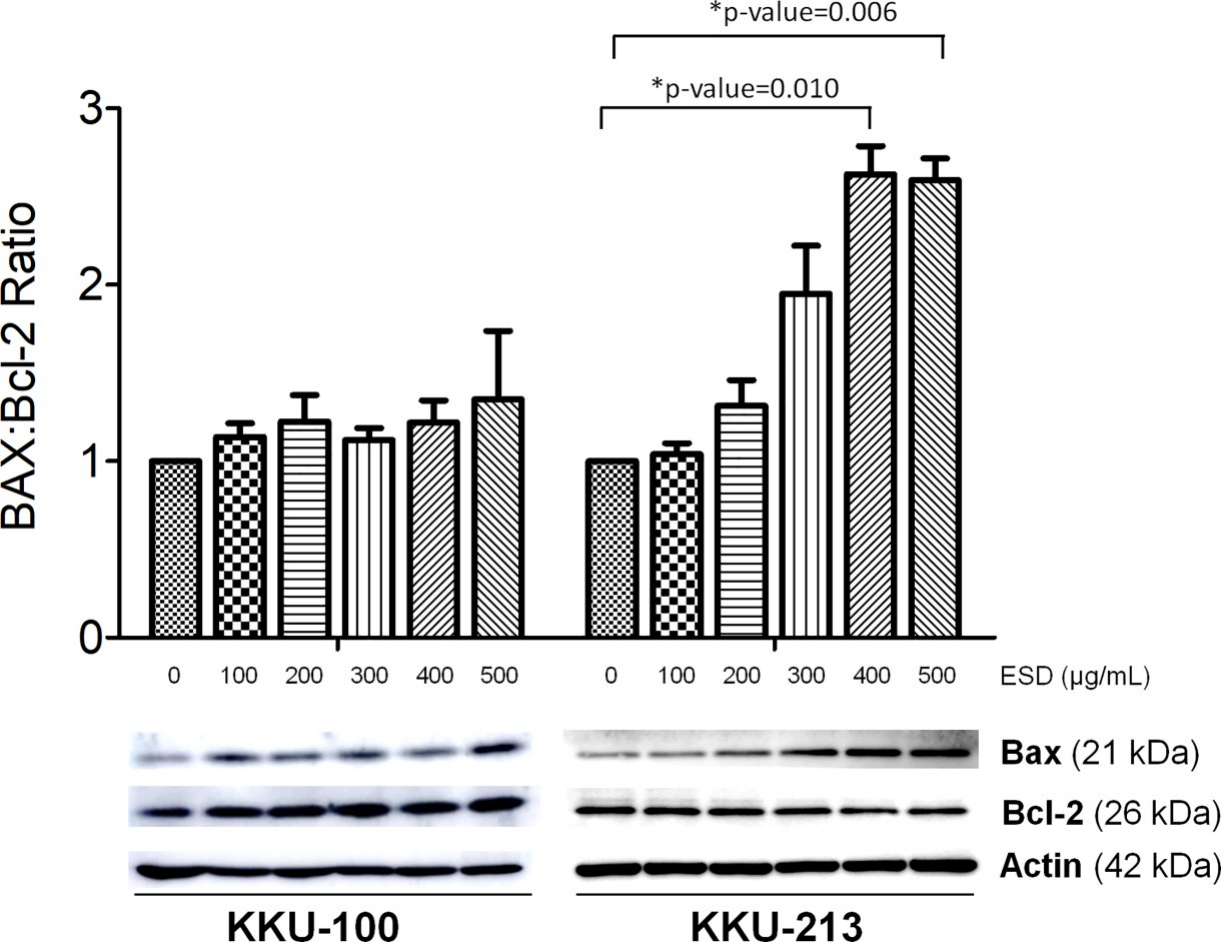
Effects of ESD on the pro-apoptotic and anti-apoptotic protein expression of CCA cell lines. Western blot analysis of BAX and Bcl-2 protein expression and the BAX/Bcl-2 protein expression ratio were determined. The data are mean ± SD of protein band intensity normalized to the intensity of β-actin from three independent experiments (* = statistical significance; p<0.05).

We examined the expression of anti-apoptosis protein, Bcl-2 and pro-apoptotic protein, BAX. Decreased Bcl-2 levels were observed in all CCA cell lines after treatment with ESD, whereas BAX protein expression increased in KKU-213. ESD, increased the ratio of BAX/Bcl-2, triggering apoptotic cell death (Fig 6).

### Identification of anticancer activity-related bioactive compounds

**Fig 7:**
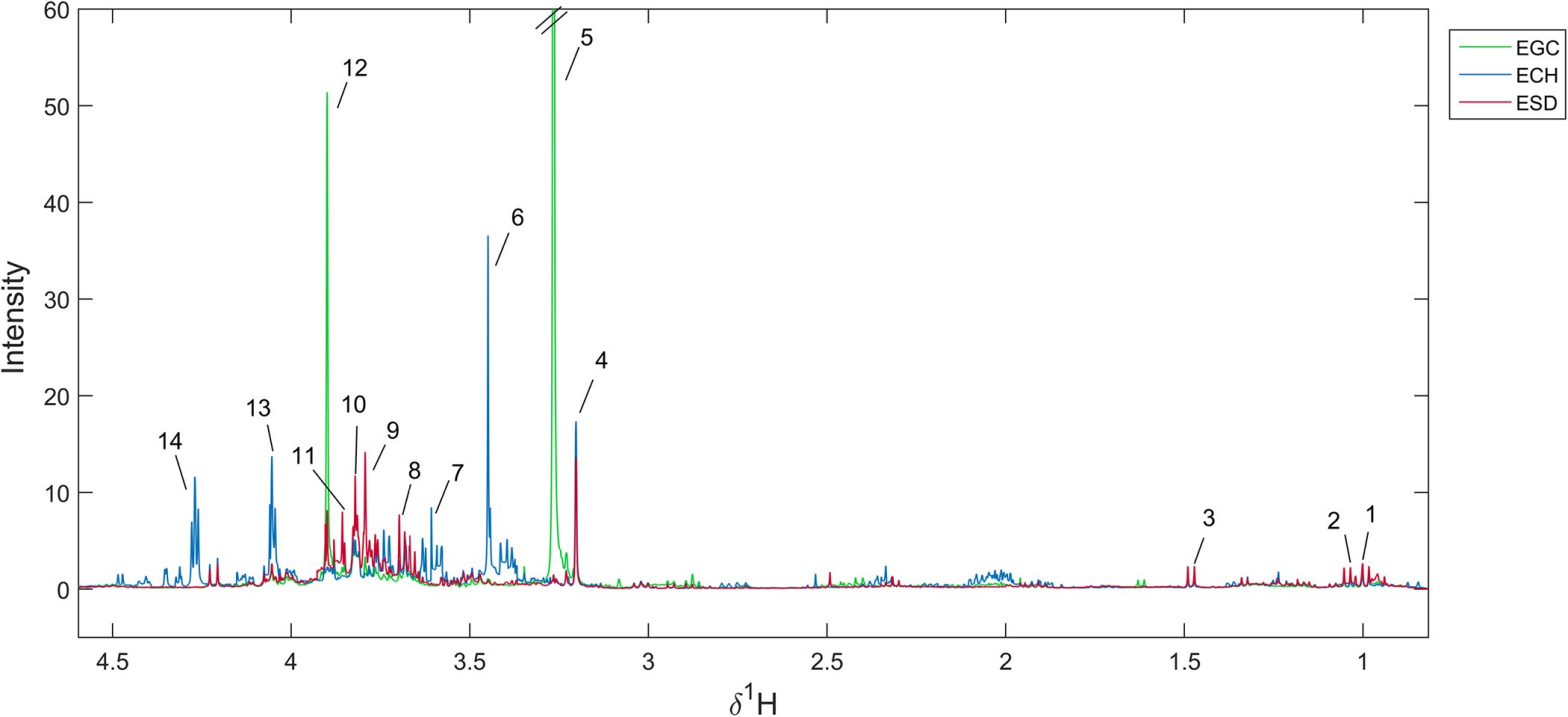
Median spectra of different crude extracts. (Key: 1 = valine; 2 = octane; 3 = alanine; 4 = xylulose; 5,12 = betaine; 6,13 = inositol; 7 = s-(5'-Adenosyl)-L-methionine; 8,9 = lignin compound 2012; 10 = glycyl-glycine; 11 = allose; 12 = coniferin, 14 = inositol phosphate).

The main metabolite compositions of each plant extract were investigated using ^1^H-NMR-based metabolic profiling (Fig 7). NMR spectra of all plant extracts showed the proton resonances at δ^1^H 1.04 (d) and 1.49 (d) ppm, indicating that all extracts contained valine and alanine, respectively. The presence of sugars (δ^1^H 3.10–4.50), including inositol, inositol pyrophosphate and xylobiose, were predominantly found in ECH, whereas xylulose and allose were observed in ESD. In addition, the predominant intensity of lignin compound 2012 (δ^1^H 3.70 (s), 3.79 (s), 4.84 (t), 5.82 (s), 6.82 (dd), 6.94 (m)) in ESD was observed. Betaine (δ^1^H 3.25 (s) and 3.89 (s)) was the dominant metabolite in EGC. It is, however, noteworthy that EGC was shown to contain coniferyl alcohol in the form of coniferin. The metabolites’ assignment is shown in S2 Table.

**Fig 8:**
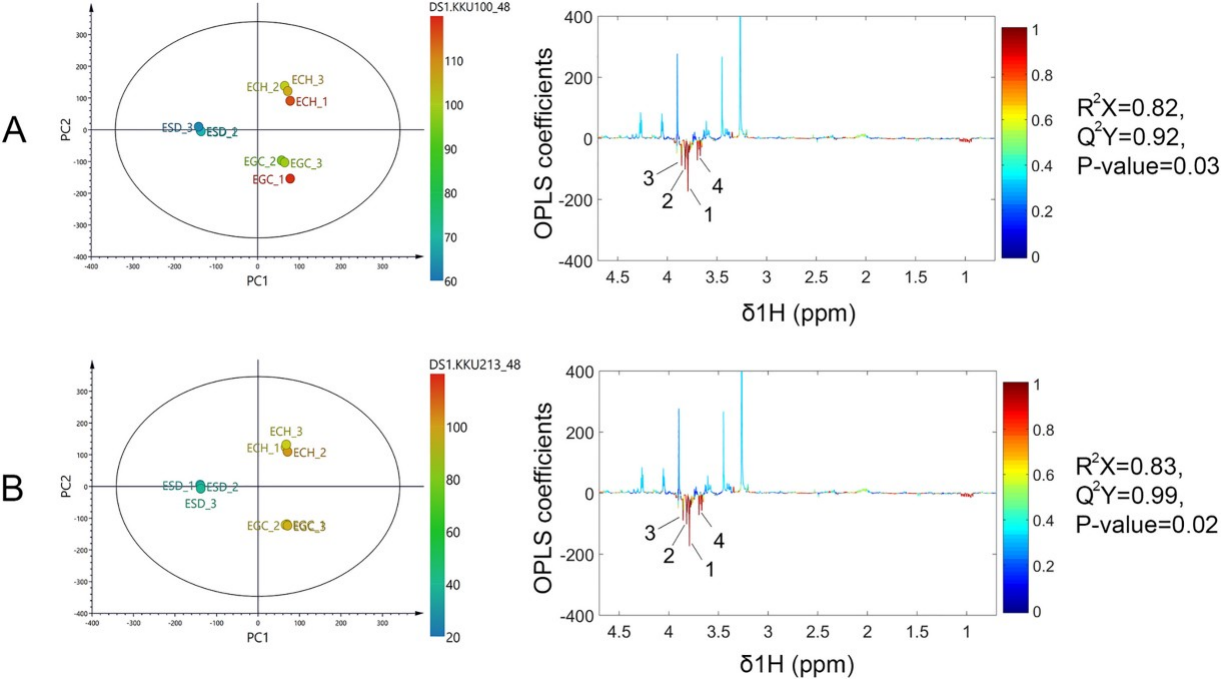
**O-PLS sores and corresponding coefficient loading plots displaying significant metabolites with cell viability on (A) KKU-100 and (B) KKU-213.** (1,4 = lignin compound 2012, 2 = glycyl-glycine, 3 = allose).

To investigate the CCA cell viability reduction-related bioactive compounds, the ^1^H NMR data sets of all plant extracts and the percentage of cell viability were analyzed using O-PLS regression analysis. The validity of the regression models was determined using a goodness of fit (R^2^X) and a goodness of prediction (Q^2^Y) of above 0.8 and 0.9, respectively. In addition, the permutation p-value, assessing the class-predictability of the model, further confirmed the validity of the O-PLS model (p < 0.05). Interestingly, the O-PLS score plots (Fig 8, left panel) indicated that ESD was inversely correlated with percentage of CCA cell viability. The coefficient loading plots of the O-PLS regression models displayed significant metabolites that correlated with either lower or higher cell viability (Fig 8, right panel). The key metabolites in ESD that were associated with lower CCA cell viability included lignin compound 2012, glycyl-glycine and allose (Fig 8). The proton signal at δ^1^H 3.79 (s), assigned as phenol lignin (CH_3_O-Ph), was the most significantly predominant peak on the NMR spectrum [33]. In addition, COSY and HMBC experiments demonstrated the β-O-4 linkage of two coniferyl acetate in the structure of lignin compound 2012 (S3 and S4 Figs). The structure of ESD-derived anticancer compound is shown in S5 Fig.

## Discussion

Medicinal plants are valuable for traditional medicine. Some parts of herbal plants, such as the roots, stems, leaves and flowers, are used for food production and treatment against some diseases as they have been shown to contain both primary and secondary metabolites. Primary metabolites are the nutrient compounds such as carbohydrates, proteins and lipids, whereas secondary metabolites include flavonoids, carotenoids and terpenoids that play vital roles in several biological activities. In the current study, the selected medicinal herbs *C*. *halicacabum*, *G*. *celosioides*, *S*. *dulcis* were extracted with 90% ethanol resulting in ECH, EGC and ESD, respectively. A previous report suggested that the crude ethanolic extraction had the highest yield of phenolics [34]. As a consequence, the presence of high phenolic content could yield remarkably high antioxidant activities [35]. Our results revealed that EGC contained the highest phenolic content and high antioxidant activity followed by ESD and ECH. The two methods of antioxidant assessments were selected to determine the extracts ability in transferring electrons (ET) or hydrogen atoms (HAT). FRAP assay is a HAT-based method, whereas, DPPH^**•**^ radical scavenging activity assay is both HAT- and ET-based [36]. In our experiment, the crude extract contains a mixture of chemical constituents. It may contain compounds that possess some metabolites with or without antioxidant property, but which could play a role in anticancer treatment. A low dose of plant extract acts as an antioxidant, decreasing the ROS level in normal cells and activating Nrf2 to bind with the antioxidant response element then inducing antioxidant enzymes expression [37]. This could be a benefit of plant phenolics for cancer chemoprevention. However, the efficacy (higher) dose usage of the plant extract plays role in inhibitory effects [12]. Recently, attention has been focused on the anticancer properties of phenolics for application in chemotherapy. This study, for the first time, provides candidate compounds derived from medicinal herbs possessing anti-CCA property.

Currently, most CCA chemotherapeutic agents have low response rates, a short median survival time and toxicity to many patients. A search for effective therapeutic agents is, therefore, needed to improve the sensitivity of treatment. To demonstrate the inhibitory effect of selected medicinal herbs, we examined CCA cell viability using the SRB assay showing that ESD greatly inhibited CCA cells from the KKU-100 and KKU-213 cell lines. The inhibitory effect of EGC on CCA cell lines was inferior to ESD and ECH. A recent study showed that an IC50 of the ethanolic and aqueous extracts of *G*. *celosioides* on HepG2 cells was higher than 250 μg/mL [14]. *C*. *halicacabum* has been reported to have an antiproliferative effect on Ehrlich ascites carcinoma (EAC) cell lines for which 200 μg/mL of the ethanolic and chloroform extracts inhibited the EAC cells by about 60 percent [38]. The presence of s-(5'-Adenosyl)-L-methionine in ECH could inhibit cancer cell growth as described by previous reports [39, 40]. However, ECH and EGC have been shown to have only a slight inhibitory effect on CCA cell growth. Only ESD had inhibitory effects on CCA cell lines by apoptosis induction. The half maximal KKU-213 growth inhibitory concentration was lower than our lowest concentration of the ESD tested (63 μg/mL). Previous studies found that betulinic acid, a triterpene isolated from *S*. *dulcis*, showed a cytotoxic effect on human carcinoma cell lines (AGS) [17]. Scopadulcis acid B, which is a diterpene, showed potential inhibition of phospholipid synthesis in skin cancer formation [18]. However, the molecular mechanistic effects of *S*. *dulcis* extract have been rarely reported.

Generally, ROS are constantly generated in aerobic cellular metabolism during oxidative phosphorylation. However, the elevation of ROS, which plays an upstream role in the activation of cell apoptosis, is a selectively strategy to inhibit cancer cells [41, 42]. Here, we describe the inhibitory effect of *S*. *dulcis* on CCA cell lines for the first time. We determined the potential of plant extracts to increase the intracellular ROS level, which might show their effectiveness as cancer cell inhibition agents through apoptosis. To investigate the apoptotic protein expression, BAX and Bcl-2 were examined in CCA cells treated with ESD. The elevated ratio of BAX/Bcl-2 confirmed that ESD triggered apoptotic cell death in KKU-213. ESD had a low sensitivity in KKU-100 on account of its *KRAS* and *TP53* mutations. Alteration of gene expression of *KRAS* and *TP53* takes place during cholangiocarcinogenesis leading to a poor prognosis and increased drug resistance in several types of cancer [43–45]. The loss of plasma membrane integrity is one of the characteristics of late apoptosis [46]. Annexin V and PI staining of human CCA cell lines after a treatment with ESD was observed using confocal microscopy. The loss of plasma membrane integrity was found in ESD treated-CCA cells in a dose-dependent manner.

Metabolomics and chemometric approaches were used in the current study to surpass traditional methods of separation, identification and evaluation of crude extracts as it is cost-effective and not as time-consuming [26]. Our results show that each plant extract carried common biological molecules, for example simple sugars and amino acids. However, distinct secondary metabolites were observed amongst the three plant extracts. Lignin, a metabolite from the ESD extract, correlates with the most significant decrease in CCA cell viability across all tested cell lines. In nature, lignins are abundant biopolymers used in the formation of a plant’s cell walls. Phenylpropanoids occupy a diverse family of polyphenols and are synthesized from phenylalanine and tyrosine [47]. Later, cinnamic acid is transformed into three types of lignols including *p*-coumaryl, coniferyl and sinapyl alcohol, which are the precursors for lignin [48] Acetylation of coniferyl alcohol produces coniferyl acetate, which is converted into eugenol, which in turn has been reported to have an anti-cancer effect through increased apoptosis [49, 50]. In this study, the proposed lignin structure consists of two coniferyl acetate linking with an ether bond, namely a β-O-4 linkage [51]. A previous study has shown that lignin fractions obtained from the conventional fractionation of *Acacia nilotica* extract had a half inhibitory concentration (IC50) on human breast cancer cell line (MCF-7) lower than 100 μg/mL [52] This is in accordance with our findings with an IC50 on CCA cell lines lower than 63 μg/mL. In addition, water soluble lignin induced apoptosis in lymphoblastic leukemia cells due to morphological changes, such as nuclear condensation and membrane blebbing observed by light microscopy [53]. This finding is in agreement with our results as ESD could induce phosphatidylserine exposure on the cell surface, detected by annexin V staining [53]. To improve anticancer drug delivery, a previous study demonstrated that lignin nanoparticles were developed to control drug release and enhance drug sensitivity against human breast cancer cells [54]. Metabolic profiling revealed greater insights into the effects of polyphenols. However, the metabolic changes and health outcomes remain a challenge [55, 56]. Therefore, the biological mechanisms of lignin underlying the anti-CCA effect should be further elucidated.

## Conclusion

Among the plants studied, ESD had the most inhibitory effect on CCA cells, with the key metabolite involved in the reduction of CCA cell viability being lignin. Annexin V and PI were present in KKU-213 cells treated with ESD. In addition, the BAX/Bcl-2 ratio was increased in a dose dependent manner. These findings suggest that KKU-213 was inhibited through apoptosis induction. Further research is, however, needed, especially on the effects of lignin and its metabolites with respect to their anti-proliferative effects.

## Supporting information

S1 Table IC50 of the studied plant extracts on CCA cells. (DOCX)

S2 Table The identification of metabolites detected in the studied plant extracts. ^1^H-NMR data are measured in ppm.
(DOCX)

S1 Fig The effect of ESD on ROS levels in KKU-100 and KKU-213. (TIF)

S2 Fig Percentage of cellular ROS positive cells in KKU-100 and KKU-213. (TIF)

S3 Fig COSY experiment. (TIF)

S4 Fig HMBC experiment. (TIF)

S5 Fig The structure of lignin compound 2012. (TIF)
