# Supplementary material for: Evaluation of anticancer potential of Thai medicinal herb extracts against cholangiocarcinoma cell lines
Source: PLoS One. 2019 May 23;14(5):e0216721. doi: 10.1371/journal.pone.0216721 (PMC6532846; doi:10.1371/journal.pone.0216721)
Supplement: S1 Table — (DOCX) [file pone.0216721.s001.docx]

**Supplementary data 1. The half maximal inhibitory concentration (IC50)**

**S1 Table** IC50 of the studied plant extracts on CCA cells.

| Cell lines | Time | IC50 (µg/mL) | | |
| --- | --- | --- | --- | --- |
|  |  | ECH | EGC | ESD |
| KKU-213 | 48 hrs | >1000 | >1000 | 161.52±35.09 |
|  | 72 hrs | >1000 | >1000 | 109.26±14.79 |
| KKU-100 | 48 hrs | >1000 | >1000 | 808.04±88.85 |
|  | 72 hrs | >1000 | >1000 | 228.67±12.67 |
