# Supplementary material for: Evaluation of anticancer potential of Thai medicinal herb extracts against cholangiocarcinoma cell lines
Source: PLoS One. 2019 May 23;14(5):e0216721. doi: 10.1371/journal.pone.0216721 (PMC6532846; doi:10.1371/journal.pone.0216721)
Supplement: S2 Table — 1H-NMR data are measured in ppm. (DOCX) [file pone.0216721.s002.docx]

**Supplementary data 3. The metabolite assignment from ^1^H NMR**

**S2 Table** The identification of metabolites detected in the studied plant extracts. ^1^H-NMR data are measured in ppm.

| Extracts | Metabolites | Chemical shift values |
| --- | --- | --- |
| ECH | Valine | δ 0.98 (d), δ 1.03 (d) |
|  | Alanine | δ 1.49 (d) |
|  | Threonine | δ 1.32 (d) |
|  | Inositol | δ 3.44 (dd), δ 3.61 (t), δ 4.04 (t) |
|  | Inositol phosphate | δ 3.44 (dd), δ 3.61 (t), δ 4.04 (t), δ 4.27 (t) |
|  | s-(5'-Adenosyl)-L-methionine | δ 2.30 (t), δ 3.07 (s), δ 3.67 (m), δ 4.30 (m) |
|  | 1,4-D-xylobiose | δ 3.43 (m), δ 3.62 (d), δ 4.05 (t) |
| EGC | Alanine | δ 1.49 (d) |
|  | Valine | δ 0.98 (d), δ 1.03 (d) |
|  | Manitol | δ 3.78 (d), δ 3.82 (dd) |
|  | Octane | δ 0.88 (s), δ 1.26 (s) |
|  | Betaine | δ 3.25 (s), δ 3.89 (s) |
|  | Xylulose | δ 3.50 (s), δ 3.60 (s), δ 3.62 (q) |
|  | Coniferin | δ 3.90 (s), δ 6.76 (d) |
| ESD | Valine | δ 0.98 (d), δ 1.03 (d) |
|  | Alanine | δ 1.49 (d) |
|  | Threonine | δ 1.32 (d) |
|  | Octane | δ 0.88 (s), δ 1.26 (s) |
|  | Lignin compound 2012 | δ 3.70 (s), 3.79 (s), δ 4.84 (t), δ 5.82 (s),  δ 6.82 (dd), 6.94 (m) |
|  | Glycyl-glycine | δ 3.82 (s), δ 3.81 (s) |
|  | Allose | δ 3.79 (m), δ 3.86 (dd) |
