# Supplementary figures and images for: Evaluation of anticancer potential of Thai medicinal herb extracts against cholangiocarcinoma cell lines
Source: PLoS One. 2019 May 23;14(5):e0216721. doi: 10.1371/journal.pone.0216721 (PMC6532846; doi:10.1371/journal.pone.0216721)

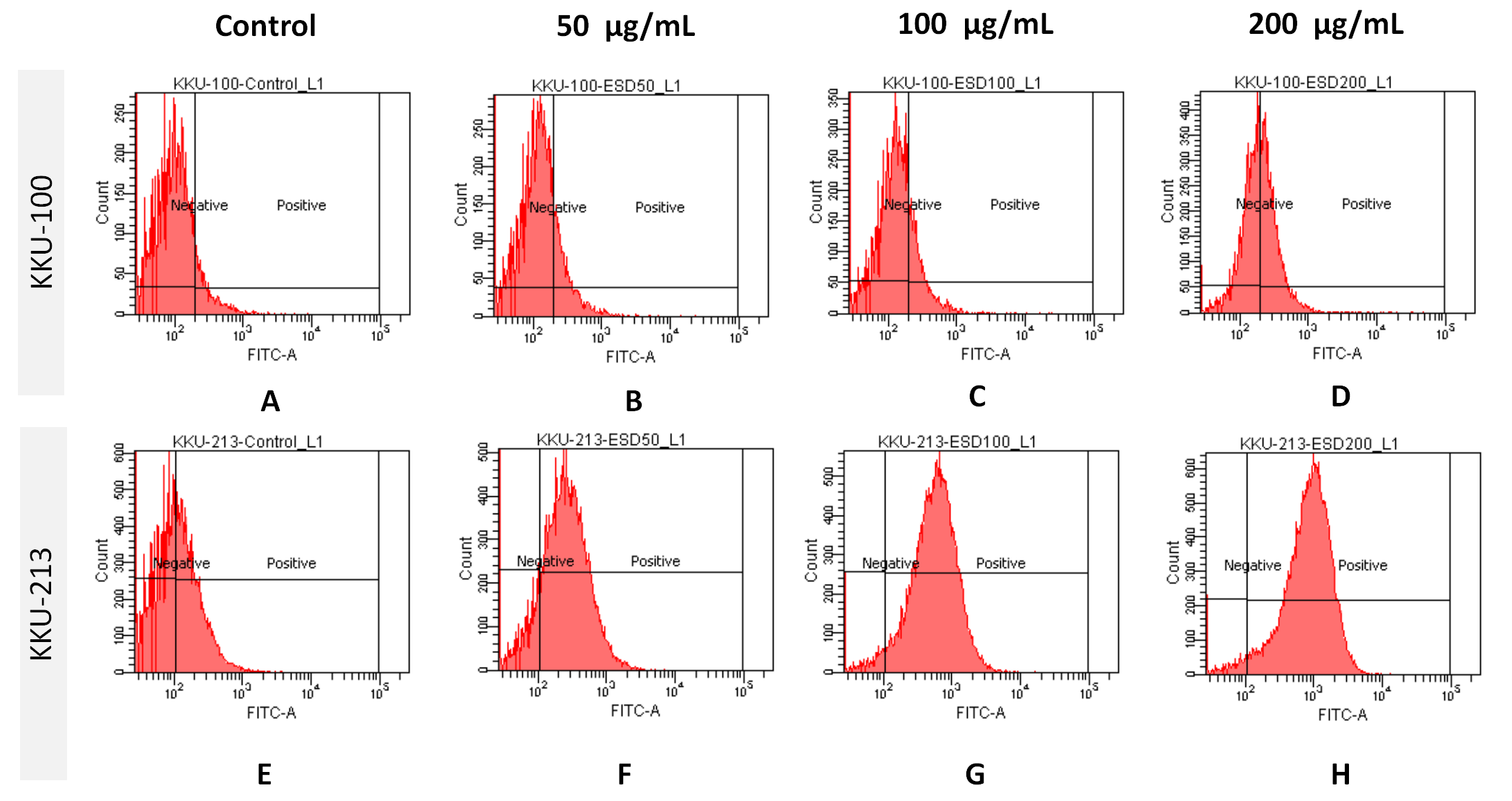

Supplement: S1 Fig — (TIF) [file pone.0216721.s003.tif]

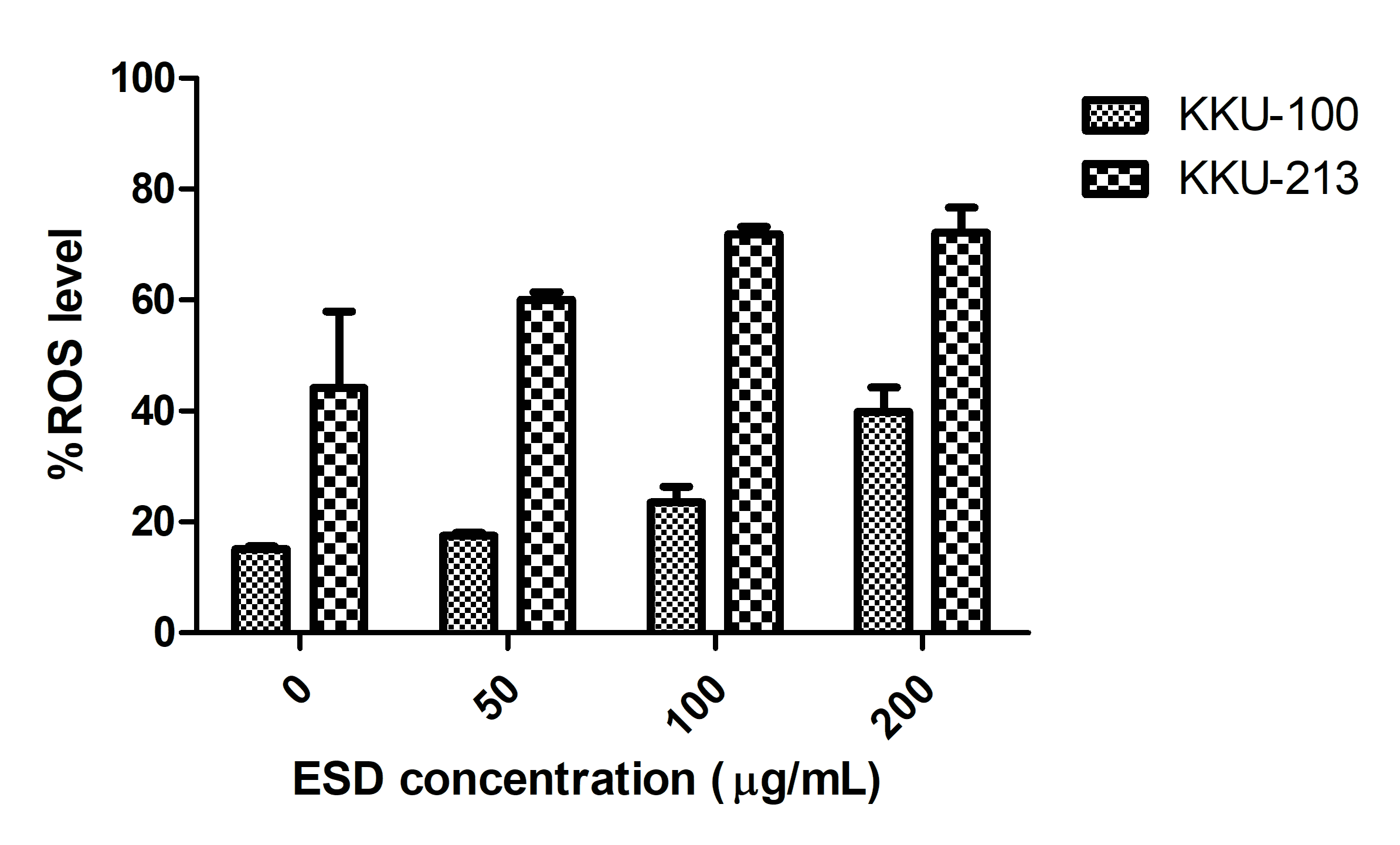

Supplement: S2 Fig — (TIF) [file pone.0216721.s004.tif]

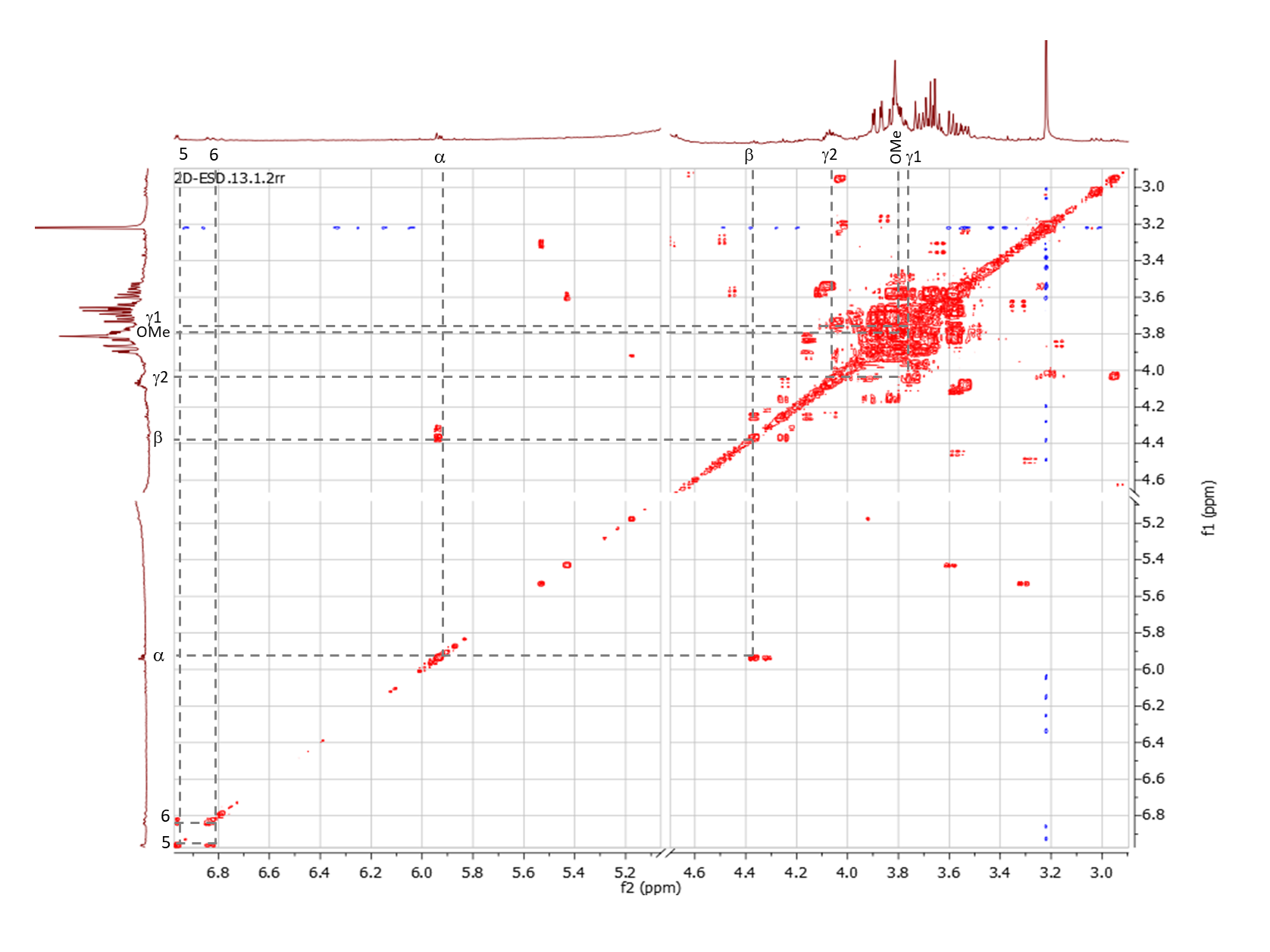

Supplement: S3 Fig — (TIF) [file pone.0216721.s005.tif]

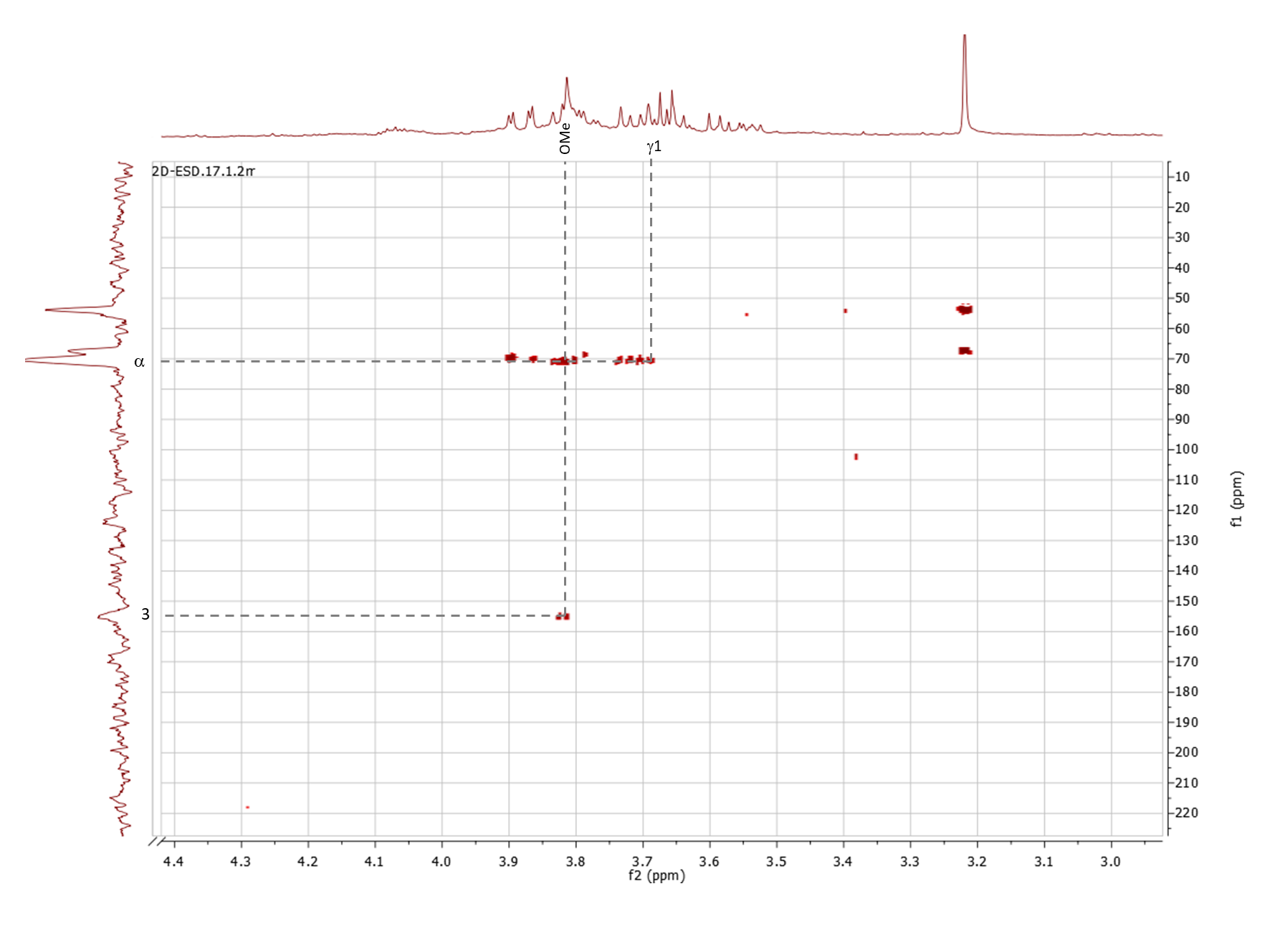

Supplement: S4 Fig — (TIF) [file pone.0216721.s006.tif]
